# Serum albumin and osmolality inhibit *Bdellovibrio bacteriovorus* predation in human serum

**DOI:** 10.1038/s41598-017-06272-2

**Published:** 2017-07-19

**Authors:** Hansol Im, Sangmo Son, Robert J. Mitchell, Cheol-Min Ghim

**Affiliations:** 1 School of Life Sciences, Ulsan National Institute of Science & Technology, 50 UNIST-gil Ulju-gun, Ulsan, 44919 Republic of Korea; 2 Department of Physics, Ulsan National Institute of Science & Technology, 50 UNIST-gil Ulju-gun, Ulsan, 44919 Republic of Korea

## Abstract

We evaluated the bactericidal activity of *Bdellovibrio bacteriovorus*, strain HD100, within blood sera against bacterial strains commonly associated with bacteremic infections, including *E. coli, Klebsiella pneumoniae* and *Salmonella enterica*. Tests show that *B. bacteriovorus* HD100 is not susceptible to serum complement or its bactericidal activity. After a two hour exposure to human sera, the prey populations decreased 15- to 7,300-fold due to the serum complement activity while, in contrast, the *B. bacteriovorus* HD100 population showed a loss of only 33%. Dot blot analyses showed that this is not due to the absence of antibodies against this predator. Predation in human serum was inhibited, though, by both the osmolality and serum albumin. The activity of *B. bacteriovorus* HD100 showed a sharp transition between 200 and 250 mOsm/kg, and was progressively reduced as the osmolality increased. Serum albumin also acted to inhibit predation by binding to and coating the predatory cells. This was confirmed via dot blot analyses and confocal microscopy. The results from both the osmolality and serum albumin tests were incorporated into a numerical model describing bacterial predation of pathogens. In conclusion, both of these factors inhibit predation and, as such, they limit its effectiveness against pathogenic prey located within sera.

## Introduction

Bacteremia is defined as the presence of bacteria within the bloodstream and is a medical condition that poses a serious concern for the patient, particularly if it leads to sepsis. Although the immune system generally has the capacity to surveil against bacteria within our bloodstream, in patients where the immune system is not functionally developed, or where it has been compromised due to illness or infection, the onset and development of bacteremia can be life-threatening^1–3^. In addition to *E. coli*, which is the most common bacterium found in community-acquired bacteremia infections, *Klebsiella*, *Pseudomonas* and *Enterobacter* are all common Gram-negative species in hospital-acquired cases^4–8^. *Salmonella*, however, is the most egregious community-acquired bacteremic pathogen in Africa, where it affects primarily young children and patients infected with AIDS^7^. A direct correlation between HIV infection and *Salmonella*-associated bacteremia is supported by several studies from various countries, including Thailand, Kenya and Spain^5, 9–11^, and by reports that found the incidence of *Salmonella*-related bacteremia is 20 to 100 times more likely in patients with a depressed immunity^12, 13^. For these patients, the mortality rates were also significantly greater when bacteremia occurred^6^.

Although antibiotics are typically used to treat bacteremic infections^14^, the spread of drug-resistance amongst human pathogens can potentially limit this route of treatment. For instance, of more than 450 *Klebsiella pneumoniae* bloodstream isolates characterized in one study, approximately 11% were resistant to carbapenems and 27.1% were resistant to ceftriaxone or ceftazidime, the third generation cephalosporins^15^. Drug-resistant strains of *E. coli* and *Salmonella* have also been isolated from patients suffering from bacteremia infections^16–19^.

Predatory bacteria, such as *Bdellovibrio bacteriovorus* HD100, present another option for treatment as they have been shown to attack and kill human pathogens^20–22^, including *E. coli*, *Salmonella* and *K. pneumoniae*, and to significantly attenuate drug-resistant bacterial populations and the genes conferring resistance^23–25^. Several groups have also shown that these predatory strains are not harmful to human cell cultures^26, 27^, but the activity of predatory bacteria against prey within the blood or sera has not been evaluated, nor has the effect of the serum complement on the viability of predatory strains. Hence, in this study, we examined the activity and viability of *B. bacteriovorus* HD100 when this bacterium was exposed to human sera.

## Results

### Serum Does Not Kill *B. bacteriovorus* HD100

**Figure 1:**
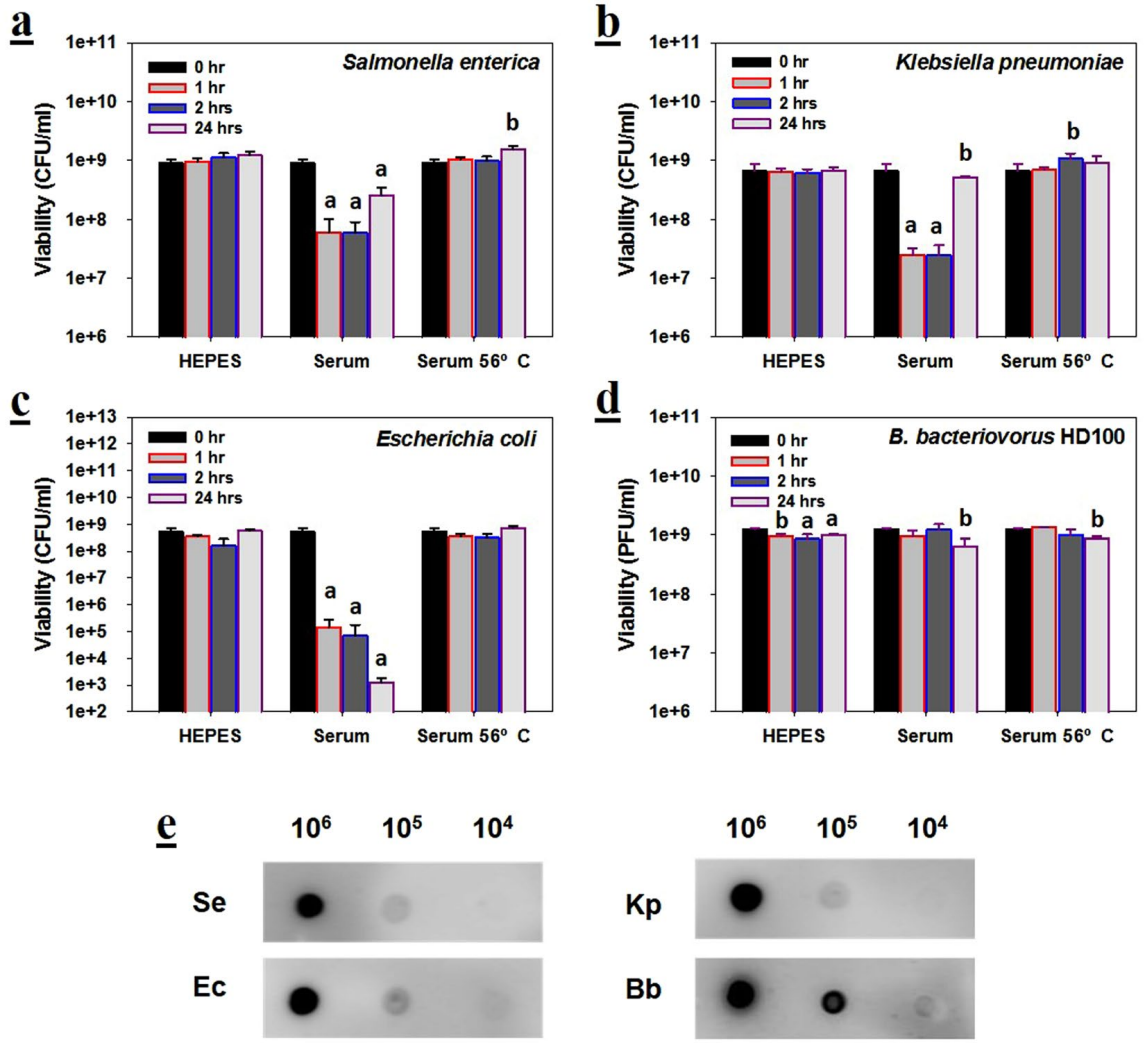
Serum complement does not significantly impact *B. bacteriovorus* HD100 viabilities. Viability of (**a**) *S. enterica*, (**b** **)** *K. pneumoniae*, (**c**) *E. coli* and (**d**) *B. bacteriovorus* HD100 in HEPES, 50% serum or 56 °C-treated serum (50%; complement inactivated) for up to 24 hours. *p* < 0.001 for a; *p* < 0.01 for b. *n* = 3. (**e**) Dot-blot results showing the serum possesses antibodies against each of the bacterial strains, particularly *B. bacteriovorus* HD100. Each spot contained between 2.5 × 10^4^ and 2.5 × 10^6^ bacteria per spot. The spots were probed using whole human serum and then with a peroxidase-conjugated anti-human goat IgG.

Initially we evaluated the impact human serum has on the viability of the bacterial strains used in this study. Both *S. enterica* and *K. pneumoniae* saw significant losses to their viabilities, *i.e*., 15- to 26-fold drop, after one hour (Fig. 1a and b) while the *E. coli* MG1655 viabilities decreased by approximately 4,000-fold in the same period (Fig. 1c). After their initial losses, however, both the *S. enterica* and *K. pneumoniae* populations recovered significantly after 24 hours while the *E. coli* MG1655 numbers continued to decrease. The results with *B. bacteriovorus* HD100 stood in contrast with its prey strains as its viability remained constant even up to 24 hours (Fig. 1d). As whole serum was used in these tests, the prey viability losses were presumably from the activity of the complement, *i.e*., a complex of serum proteins which act together to kill bacteria by forming pores in their membranes^28, 29^.

To confirm that the complement was responsible, it was inactivated by treating the serum at 56 °C and we found that the viabilities remained steady for all four strains (Fig. 1a–d). Activation of the complement requires an antibody to bind its antigen on the surface of the bacterium^30^, which leads to cascade event culminating in the formation of the pores. The lack of a clear complement activity against *B. bacteriovorus* in Fig. 1d suggested that few or no antibodies against this bacterium existed in the serum. A dot blot analysis with whole predatory cells proved this was not the case since *B. bacteriovorus* gave the strongest responses amongst all four strains (Fig. 1e).

### High Serum Osmolality Negatively Impacts Predation

**Figure 2:**
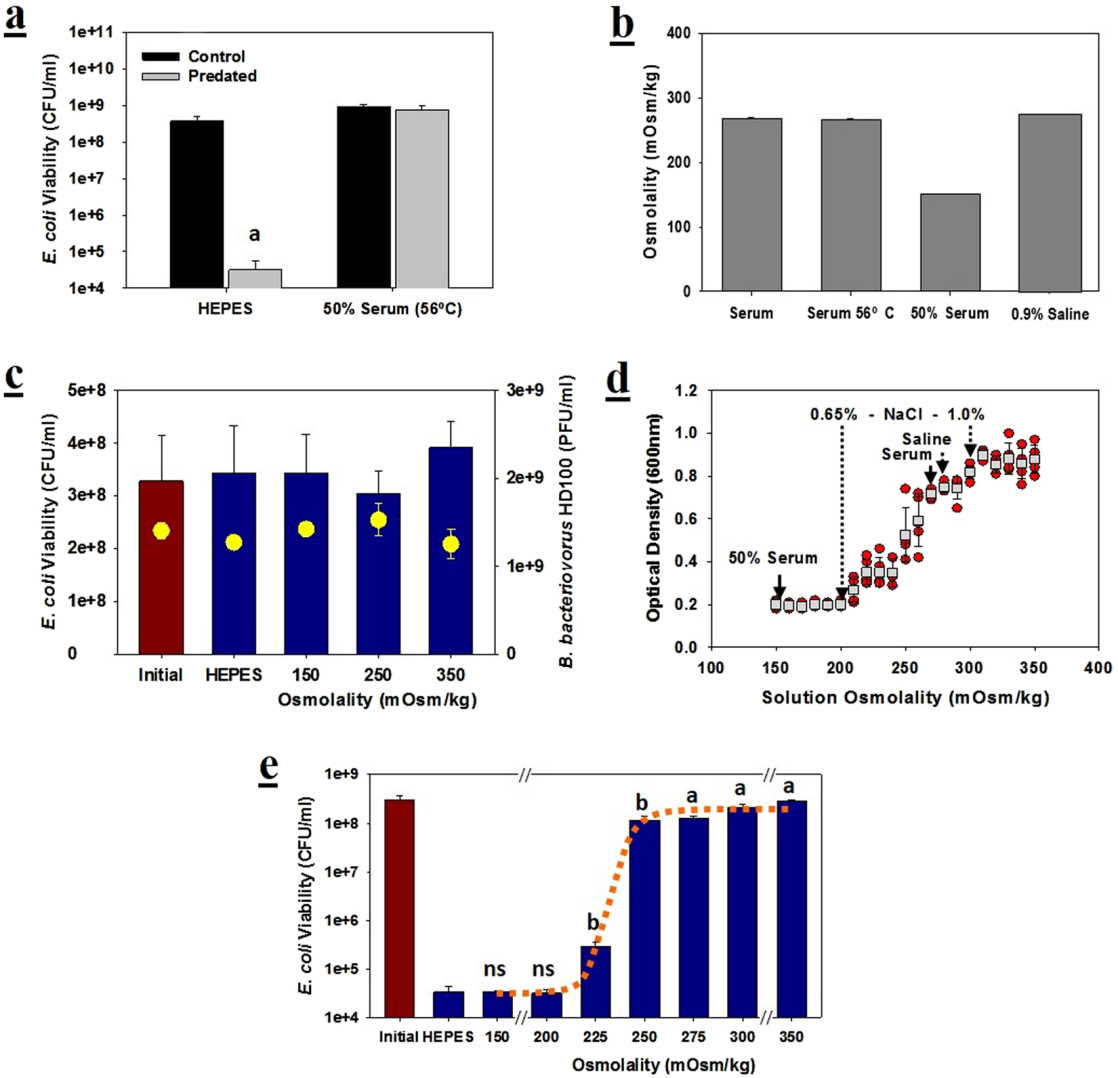
Predation is significantly inhibited by elevated osmolality. (**a**) Predation of *E. coli* MG1655 is inhibited in 50% human serum. The experiment was performed for 24 hours using either HEPES or heat-treated serum with an initial predator-to-prey ratio (PPR) of approximately 0.05. The *E. coli* viabilities were measured after 24 hours. *n* = 3. (**b**) Osmolality of the human serum samples used. The osmolality of whole serum (heat-treated and untreated) was determined alongside that of the 50% serum used in the previous experiments. For comparison, a 0.9% saline solution was also tested. *n* = 3. (**c** ) The predator and prey viabilities were not negatively impacted by the osmolalities tested. The *E. coli* viabilities are shown as bars and those for *B. bacteriovorus* HD100 are presented as yellow dots. *n* = 3. (**d**) The predatory activity of *B. bacteriovorus* HD100 is reduced as the osmolality increases. The results shown are the optical densities of the predated culture after 24 hours (red dots) and the averages from five independent tests (white squares) with their corresponding standard deviations. For reference, the osmolalities of several saline solutions and human serum are indicated. The initial PPR was approximately 0.05. *n* = 5. (**e**) *E. coli* viability based upon the osmolality. With the initial PPR of approximately 0.05, the viabilities of the prey were determined after 24 hours. The significance was determined by comparing the viabilities obtained for each osmolality values with those in the HEPES buffer. *p* < 0.01 for a; *p* < 0.05 for b. *n* = 3. The dotted orange line laid over the histogram was obtained from the numerical simulations. See Materials and Methods.

As the viability *per se* of *B. bacteriovorus* HD100 was not negatively impacted by the serum complement, it was presumed that this predator could be active in sera. However, as shown in Fig. 2a, predation of *E. coli* MG1655 did not occur in blood serum.

This lack of activity was initially considered due to the osmolality of the 50% serum solution used, which was 150 mOsm/kg (Fig. 2b). By comparison, the osmolality of whole serum and a 0.9% saline solution were 268 and 278 mOsm/kg, respectively. Treatment of the serum at 56 °C to inactivate the complement did not change this value (Fig. 2b). Consequently, the ability of *B. bacteriovorus* HD100 to predate on *E. col*i MG1655 in prepared saline solutions spanning this range of values, *i.e*., between 150 and 350 mOsm/kg, was tested. The optical density and prey viability in 24 hour are given in Fig. 2c and d, respectively. It is clear from both of these figures that the predator is inhibited by the higher osmolalities tested. The concentration range where the predators shifted from an active to an inactive state was narrow, spanning only 50 mM, or from 0.65% to 1% (w:v) NaCl. Although both Fig. 2c and d show that whole serum, with an osmolality of 268 mOsm/kg, should significantly inhibit predation, the serum used in Fig. 2a was diluted (50%) and had an osmolality that was only 150 mOsm/kg. As this value is well within the permissible range of osmolality, it is highly possible that other factors than osmolality of the serum also inhibits *B. bacteriovorus* HD100.

To elucidate the dynamic of this sharp sigmoidal transition, we conducted numerical experiments on the predator-prey model that incorporates the culture-media osmolality as a determining factor of predation rate. The prey viability obtained from the simulations closely reproduced the experimental data as shown in Fig. 2e. A key ingredient of the descriptive mathematical model was the strongly “cooperative” action of the solute particles on the predator’s capability to predate. Though the detailed molecular mechanism is yet to be revealed, it is strongly suggestive of a critical osmolality above which the predation is almost completely inhibited.

### Serum Albumin Coats the Surface of *B. bacteriovorus* and Blocks Predation

**Figure 3:**
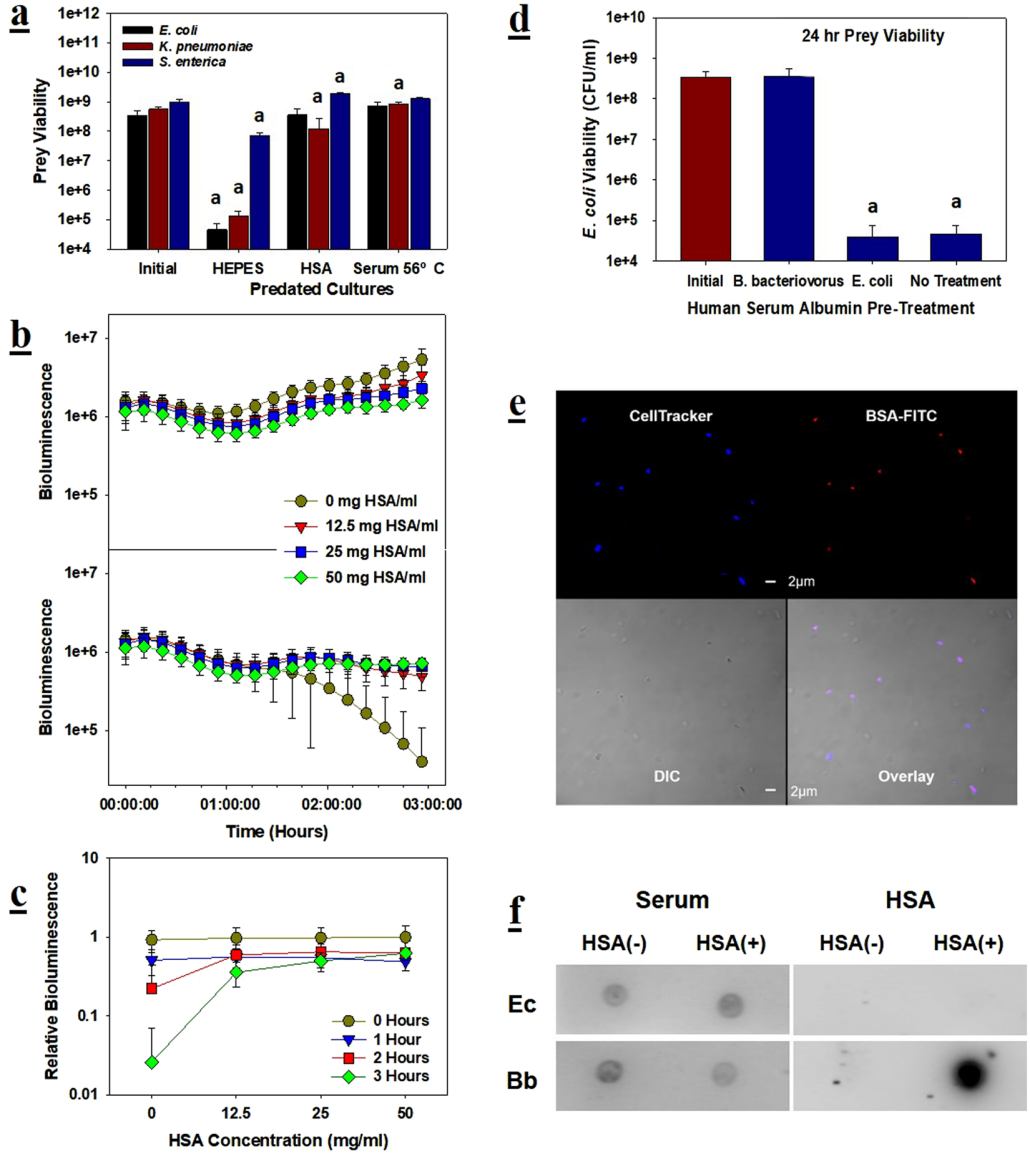
Serum albumin binds to and prevents the predator from attacking its prey. (**a**) The addition of human serum albumin (HSA) to HEPES buffer at a physiologically relevant concentration of 50 mg/ml significantly inhibits predation of all three prey strains. For comparison, predation tests were also attempted in heat-treated serum. The initial PPR was approximately 0.05 and the significance was determined by comparing the viabilities obtained after 24 hours with the initial values. *p* < 0.001 for a; *p* < 0.01 for b. *n* = 3. (**b**) Serum albumin prohibits predation of a bioluminescent *E. coli* prey strain. The bioluminescence (BL) from the prey strain was monitored over time while being predated upon in the presence of HSA. The upper panel is the control results, i.e., without *B. bacteriovorus* HD100 addition, and the bottom panel is with the predator added. The PPR was approximately 9.7 for these experiments. *n* = 9. (**c** **)** Relative bioluminescence (RBL) results calculated using the data in (**b**). The RBLs were calculated by comparing the BL from the predated sample with that of its control (unpredated) every hour and the data plotted. The results show the similar levels of inhibition obtained with each of the HSA concentrations tested. *n* = 9. (**d**) Pretreatment of *B. bacteriovorus* HD100 with serum albumin is sufficient to block predation. The predator or its prey (*E. coli*) was pretreated by incubating it for one hour in a 50 mg/ml HSA solution made with HEPES buffer. Afterwards, the predator (or prey) were pelleted, washed and mixed with fresh prey (or predator) and the predatory activity determined after 24 hours. For comparison, samples containing untreated predator and prey cells were also tested. The significance was determined by comparing the viabilities obtained after 24 hours with the initial values. *p* < 0.001 for a. *n* = 3. (**e**) Confocal images of *B. bacteriovorus* HD100 after exposure to FITC-labeled BSA. *B. bacteriovorus* HD100 cells in HEPES buffer were incubated with the Cell Tracker live stain for 30 minutes at 30 °C to produce fluorescent predatory cells. After pelleting and washing the cells, they were exposed to FITC-labeled BSA (10 mg/ml) for one hour at 30 °C and 250 rpm. The cells were washed again to remove any free or loosely bound BSA molecules before being imaged using a laser confocal microscope (LX-700, Olympus, USA). This image shows the BSA molecules (red) bound to the predatory cells (blue). Both of the scale bars in the middle indicate 2 *μ*m. (**f**) Dot blot results showing human serum albumin is biding to *B. bacteriovorus* HD100. Approximately 2.5 × 10^5^ *B. bacteriovorus* HD100 or *E. coli* were exposed to 50 mg/ml HSA, before being pelleted, washed and then spotted on the nylon membrane. The left-side blots were obtained as in Fig. 1e. The right-side blots were obtained using an anti-HSA rabbit IgG followed by a HRP-conjugated anti-rabbit goat IgG. Although tests with a prey (*E. coli*) were performed in parallel, only the predator exposed to HSA was detected.

Human serum albumin (HSA) is the most abundant protein found in human sera, present at a concentration that is typically between 35 and 53 mg/ml^31, 32^, and is known to bind to many factors, including several Gram-positive bacteria^33–35^. Thus, we tested its impact on the activity of *B. bacteriovorus* HD100 with all three prey strains (Fig. 3a). For comparison, tests were also performed in HEPES and in 56 °C-treated serum. While all three prey strains were attacked by *B. bacteriovorus* HD100 in HEPES, their populations decreasing between 1.2 and 3.8-log, performing the tests in 56 °C-treated serum prevented this. Similar results were seen with *E. coli* and *S. enterica* when HSA was added. The viability of *K. pneumoniae* decreased slightly, but significantly (4.5-fold), when predated on in the HSA media.

HSA and its impacts on predation were studied further using a bioluminescent *E. coli* as the prey (Fig. 3b and c). For these tests, HSA concentrations from 12.5 to 50 mg/ml were tested. The results show that predation was inhibited at each of these concentrations. This is particularly evident in Fig. 3c, where the relative bioluminescence is provided. Moreover, similar results were obtained with bovine serum albumin (BSA), implying that this is a general characteristic of albumins (Supplementary Figure 1). Additional experiments indicate HSA inhibits predation by binding to *B. bacteriovorus* HD100 (Fig. 3d). For these tests, either the predator or *E. coli* were pre-treated with 50 mg/ml HSA and washed prior to initiating predation. The results in Fig. 3d show pre-treatment of *B. bacteriovorus* HD100 was sufficient to inhibit predation. In contrast, pre-treating the prey with HSA did not protect it from the predator (Fig. 3d).

Using a FITC-labeled BSA, we established that albumin proteins bind to and coat this predatory bacterium (Fig. 3f). Supplementary Figure 2 is a close-up image of the *B. bacteriovorus* HD100 flagella and shows FITC-BSA also coated the flagella of this predator. This, however, did not impede the ability of *B. bacteriovorus* HD100 to swim. As shown in the Supplementary Video, after treating *B. bacteriovorus* HD100 with 50 mg/ml HSA the average swim speed was 24.7 ± 13.1 *μ*m/s while that of the untreated controls was 25.1 ± 17.5 *μ*m/s (sample size: *n* = 30).

**Figure 4:**
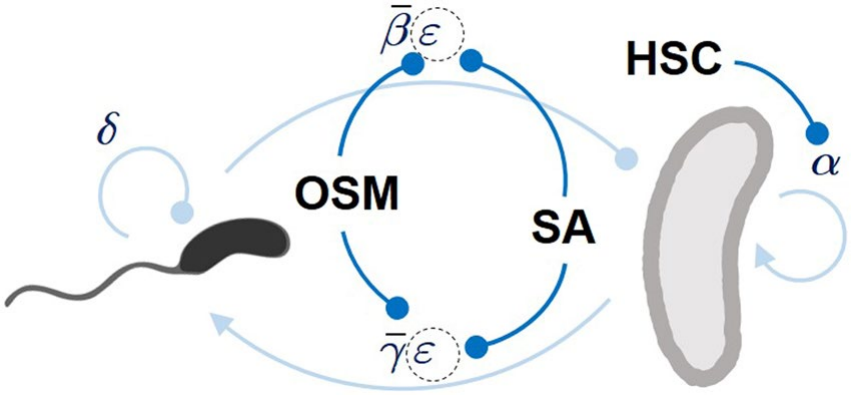
Schematic diagram of ecological interactions in a microbial predator-prey system. Environmental factors affecting the ecological interaction between the predator (left) and the prey (right). OSM: high osmolarity of the medium, SA: serum albumin, HSC: human serum complement. Arrows indicate a positive contribution to the population growth while the filled circles do a negative contribution. The lighter blue lines represent the Lotka-Volterra model while the darker blue indicate negative impacts of the environmental factors. The changes in OSM and SA is reflected in the parameter *ε* but not in $\bar{\beta }$ or $\bar{\gamma }$.

Binding of HSA to the the predatory bacterium was also confirmed by dot-blot analyses using anti-human HSA antibodies (Fig. 3f). As shown in the blot, the antibodies reacted with only the HSA-exposed predators and not the *E. coli* prey or unexposed predatory cells. The impact of HSA on the dynamics of bacterial predation, despite possible differences in the underlying molecular mechanism, can be accounted for in the same manner as the effect of osmolality as this serum protein clearly has a negative impact on predation, which is almost negligible at the threshold concentration of ca. 15 mg/ml. These were combined to further develop our modified Lotka-Volterra model to describe bacterial predation^36^, as illustrated in Fig. 4.

## Discussion

One defense that the human body has against bacterial cells present in the serum is the complement, *i.e*., a group of proteins that work in conjunction with each other to kill bacterial cells. Once activated, the complement proteins enter a cascade that culminates in the formation of a membrane pore that allows non-specific transport across the membrane, such as allowing cellular ATP to seep out. When the prey strains were exposed to human sera, all three were susceptible to complement killing as evidenced by reduced viabilities after only an hour, while heat-treating the sera at 56 °C denatured the complement system and effectively blocked serum killing (Fig. 1). *B. bacteriovorus* HD100, however, was resistant to serum complement killing. The dot blot results in Fig. 1d demonstrate some antibodies within human serum recognize this predatory strain or its surface constituents. Given *Bdellovibrio* cells are found within the human gut^37^, the presence of antibodies against this bacterium or its surface proteins is clearly feasible, though the reactivity seen in Fig. 1e may result from antibodies that bind to common bacterial surface proteins, such as porins. The clear presence of antibodies against *B. bacteriovorus* HD100 without a concomitant loss of viability, however, advanced the idea that this predator is resistant to complement. One possible explanation for this is the unique lipid A molecules present in this microorganism, where the phosphate groups have been replaced by mannose residues, effectively making the lipid A neutral in electric charge^38^. According to Chonn *et al*. (1991), the charge of the membrane is an important factor to consider since positively- and negatively-charged membranes activate serum complement while net neutral membranes do not^39^.

Although *B. bacteriovorus* HD100 was not killed by complement, its predatory activity in serum was still significantly constrained by two additional factors: the osmolality and albumin. *B. bacteriovorus* HD100 can easily predate on these prey strains in low-osmolality media, such as dilute nutrient broth (DNB) or HEPES buffer^40, 41^. Using various saline preparations, therefore, we evaluated the activity of *B. bacteriovorus* HD100 against *E. coli* MG1655 at different osmolalities. While osmolalities as high as 350 mOsm/kg did not significantly reduce the viability of the predator or its prey, the higher values tested did inhibit predation. The NaCl concentration range where the predators shifted from an active (200 mOsm/kg) to an inactive state (250 mOsm/kg) was small and spanned only 25 mM, or an increase from 0.65% to 0.82% NaCl. When the osmolality of the solution was 200 mOsm/kg or lower, predation occurred readily, as evidenced by the similar losses in both the culture optical densities and the prey viabilities. As the osmolality increased, predation was progressively inhibited and only occurred mildly when the media osmolality was 250 mOsm/kg. In this media (0.82% NaCl), the *E. coli* MG1655 population loss was approximately three-fold while, with slightly less salt (0.65% NaCl), the loss was 10,000-fold. As the osmolality of blood serum is reported to be 285 to 295 mOsm/kg^42^, the findings here imply that the predation in blood sera should be severely inhibited due to the osmolality alone. However, we could not demonstrate this directly in blood sera since predation is also inhibited by serum albumin.

Albumin is renowned for binding many different chemicals and even Gram-positive bacteria, but this study is the first reported demonstration that this serum protein also binds to the Gram-negative *B. bacteriovorus* HD100. The impact of HSA was specifically against this predator, as illustrated in Fig. 3d and f. Once coated, the predator was no longer capable of attacking the *E. coli* prey, even after being washed, while treatment of the prey had no impact. Moreover, antibodies specific for HSA reacted with only the treated predatory cells, verifying that HSA binds to *B. bacteriovorus* but not *E. coli*. Based upon the tests with FITC-labeled BSA, albumin coats the entire bacterium, including its flagella. Although the flagellum in most Gram-negative bacteria is attached to and protrudes from the outer membrane, *B. bacteriovorus* is one of a small group of microorganisms that sheath their flagella with their outer membrane, a grouping which includes *Vibrio cholerae*, *Helicobacter pylori* and *Brucella melitensis* ^43–46^.

As such, the clear demonstration of FITC-labeled albumin proteins on the flagella only serve to prove HSA is adhering to the outer membrane of *B. bacteriovorus* HD100. This finding raised the question if the presence of serum albumin on the flagellum acts to reduce the speed of *B. bacteriovorus*. This was not the case, however, as both the control and treated predatory cells were similarly active and motile (Supplementary Video).

Another intriguing point in the microbial population dynamics observed from this study is the “switch-like” transition behavior in predation activity. In general, sharp transitions in dynamical systems are commonly attributed to cooperativity or some sort of synergistic effect. It suggests a cooperative binding of HSA to the predator cells and, by the same token, cooperative accumulation or uptake of certain solutes^47^.

In conclusion, predation of bacterial pathogens in human serum does not seem feasible. Although our results show that serum complement is not active against *B. bacteriovorus* HD100, additional factors were found to work against this predator and its bactericidal activity will severely limit the potential of predatory bacteria to treat pathogens within the bloodstream.

## Materials and Methods

### Bacterial strains and cultures

The bacterial prey strains used in this study include *E. coli* MG1655, *Salmonella enterica* KACC 11595, and a clinical isolate of *Klebsiella pneumoniae*, as well as the predatory bacterium *Bdellovibrio bacteriovorus* HD100. The clinical isolate was originally obtained from a urinary catheter at the Pusan National University Hospital. Each of the prey was cultured in Luria broth (LB) media and prepared for the predation tests as described previously^25, 48^. Briefly, cultivation of *B. bacteriovorus* HD100 was performed using *E. coli* MG1655 as the prey. An overnight culture of *E. coli* MG1655/pUCDK grown in LB media was centrifuged (4000 *g* for 15 min) and resuspended in HEPES buffer (25 mM with 3 mM MgCl _2_ and 2 mM CaCl _2_, pH 7.2) at an optical density (OD, 600 nm) of 1.0. The OD was measured using an Eppendorf Biophotometer Plus.

After preparing the prey, a filtered (0.45 *μ*m) culture of the predator was added at a 1:100 dilution, giving a predator-to-prey ratio (PPR) between 0.02–0.05. The predation culture was then incubated overnight at 30 °C and 250 rpm. The OD of the predated culture after 24 hours was typically near or below 0.2.

### Human serum viability assays

Human serum (Male, AB plasma; Cat #H522) was purchased from Sigma-Aldrich (USA). To measure the impact of the human serum on the viability of the prey bacterial strains, overnight cultures of each strain were centrifuged (4,000 *g* for 15 min) and resuspended in HEPES buffer (25 mM, pH 7.2) at an OD of 2.0. This corresponds to 5.1 × 10^8^–2.2 × 10^9^ colony forming units (CFU) per ml, depending on the strain. The preparations were then mixed 1:1 (v:v) with either untreated human serum or human serum that was incubated at 56 °C to inactivate the complement. These samples were incubated at 30 °C for up to 24 hours, with aliquots taken immediately and after 1, 2 and 24 hours to measure the prey viabilities (CFU/ml). For *B. bacteriovorus* HD100, the predatory culture was prepared using *E. coli* MG1655 as described above, filtered (0.45 *μ*m) and centrifuged (16,000 *g* for 5 min). The pelleted predatory cells were resuspended in an equal volume of fresh HEPES buffer and this was mixed 1:1 (v:v) with the human serum samples. The viability of the predator cells was measured at the same time points as the prey cells. For this, aliquots were diluted in HEPES buffer and used to prepare top agar plates as described previously^25^, with *E. coli* MG1655 as the prey.

### Dot blot analyses

Each of the bacterial strains were pelleted and resuspended in HEPES buffer to a concentration of 8.9 ± 1.3 × 10^8^ CFU/ml for *S. enterica*, 6.2 ± 0.7 × 10^8^ CFU/ml for *E. coli*, 8.7 ± 1.2 × 10^8^ CFU/ml for *K. pneumoniae* and 9.7 ± 1.3 × 10^8^ PFU/ml for *B. bacteriovorus* HD100. This was serially diluted 10- and 100-fold and 2 *μ*l was spotted from each of these preparations onto a nitrocellulose membrane and dried for 15 minutes at 37 °C. The membrane was then blocked using a 5% skim milk Tris-buffered saline (TBS) solution for one hour at room temperature. After washing the blot three times for five minutes each with TBS containing 0.05% tween-20 (TBS-T), the membrane was probed with a 5% skim milk-TBS solution with human serum added (1:15 dilution). After an hour of incubation, the membrane was again washed three times with TBS-T and the probed with an HRP-conjugated goat anti-human IgG antibody (Sigma-Aldrich, USA) for one hour in a 5% skim milk-TBS solution. The membrane was washed twice more with TBS-T and a final time with TBS.

To visualize the dots, the membrane was either treated with a TMB solution (Thermo-Fisher Scientific, USA) or with a chemiluminescent substrate (Pierce, USA). For the HSA-binding assays, the concentrations of *E. coli* and *B. bacteriovorus* HD100 were 9.6 ± 1.8 × 10^8^ CFU/ml and 12.7 ± 0.9 × 10^8^ PFU/ml, respectively. These solutions were diluted tenfold and 2 *μ*l was spotted onto the membranes. The protocol for probing the blots was the same as above except that the 1° (rabbit anti-HSA) and 2° (goat anti-rabbit, HRP-conjugated) antibodies were both used at a 1:20,000 dilution.

### Osmolality assays

The osmolality of the various samples was measured using an Advanced 3220 Freezing-Point Osmometer (Advanced Instruments, USA) according to the manufacturer’s suggested protocol. To adjust the osmolality of the samples, concentrated NaCl solutions were mixed 1:1 (v:v) with the HEPES preparations. To test the impact of the osmolality on predation, prey preparations in HEPES buffer (OD of 2.0) were mixed 1:1 (v:v) with the appropriate NaCl solution. To this, the predatory filtrate was added (1:100 dilution). These samples were then incubated for 24 hours at 30 °C with agitation (250 rpm), after which the culture OD and prey viability were determined.

### Human serum albumin inhibition of predation

The impact of human serum albumin (HSA) on predation was tested using fresh solutions of 100 mg/ml HSA prepared in HEPES buffer. In parallel, the prey cultures were prepared as above in HEPES buffer and at an OD of 2.0. These two solutions were mixed 1:1 (v:v). To the test culture, the predatory filtrate was added (1:100 dilution). For a positive control, the prey culture was prepared to an OD of 1.0 using HEPES buffer alone and the same amount of predatory culture was added. The samples were incubated for 24 hours at 30 °C with agitation (250 rpm), after which the prey viability (CFU) was determined.

### Bioluminescence assay

The predation activity in the presence of HSA was measured using a slight modification of the bioluminescent prey protocol described previously^36^. Briefly, the bioluminescent prey, *E. coli* MG1655/pUCDK, was resuspended to an OD of 0.05 in HEPES buffer containing HSA (100 mg/ml) and 200 *μ*l was aliquoted into the first row of a 96-well plate (Greiner, USA). In parallel, the prey was also resuspended to an OD of 0.05 in HEPES buffer and 100 *μ*l was aliquoted into the remaining wells of the plate. From the first row, 100 *μ*l was serially diluted into the lower rows, producing a range of HSA concentrations while maintaining the prey concentration. Finally, to each well, 100 *μ*l of a diluted predatory filtrate was added to give a PPR of 10. The plate was incubated at 30 °C within a GloMax Plate Reader (Promega) and the bioluminescence (BL) measured at set times for up to four hours. The same protocol was used for the bovine serum albumin tests.

### Human serum albumin pre-treatment

Cultures of either *E. coli* MG1655 or *B. bacteriovorus* HD100 were mixed with an equal volume of a HEPES solution containing 100 mg/ml HSA. The cells were incubated in this solution for one hour at 30 °C before being pelleted (16,000 *g* for 5 min), washed and resuspended in fresh HEPES buffer. The impact of pre-treating the predator or prey was then determined using the same protocol as described above.

### Predator-prey model and numerical simulations

A conventional Lotka-Volterra model^49^ has been modified to incorporate the finite availability of the resources in culture media. The resulting predator-prey model is the following system of ordinary differential equations

1 $$\{\begin{array}{rcl}\frac{{\rm{d}}x}{{\rm{d}}t} & = & \alpha x\,(1-\frac{x}{\kappa })-\beta xy\\ \frac{{\rm{d}}y}{{\rm{d}}t} & = & \gamma xy-\delta y\end{array},$$

where *x* and *y* respectively denotes the population size of the live prey and predator cells. The term proportional to *α* reflects the logistic growth of the prey population due to the same-species competition for the limited resources in a culture medium, which is captured by the carrying capacity *κ*. The two terms proportional to the product *xy* represent the negative (positive) impact of predation events on the prey (predator) population. Finally, the term proportional to *δ* stems from the natural decay of predator cells when the prey cells are depleted (*x* = 0). The rate of predation and its impact on each population *β* and *γ* has been inferred from the previous experimental study^45^ for the average mobility of *Bdellovibrio* HD100 strain and the estimated probability of successful predation. To incorporate the effects of complement factors, *α* has been set inversely proportional to the concentration of serum complement protein. Likewise, the impacts of medium osmolality and serum albumin are reflected in the rate parameters *β* and *γ* while keeping the ratio *β*/*γ* intact. See Fig. 4.

## Electronic supplementary material

Supplementary Video 1

Supplementary Video 2
